# Extracellular Vesicles and DAMPs in Cancer: A Mini-Review

**DOI:** 10.3389/fimmu.2021.740548

**Published:** 2021-10-15

**Authors:** Nadiah Abu, Nurul Ainaa Adilah Rus Bakarurraini, Siti Nurmi Nasir

**Affiliations:** UKM Medical Molecular Biology Institute (UMBI), Universiti Kebangsaan Malaysia Medical Centre, Kuala Lumpur, Malaysia

**Keywords:** exosome, TLR, PRR, tumor microenvironment, cancer

## Abstract

Certain cancer therapy has been shown to induce immunogenic cell death in cancer cells and may promote tumor progression instead. The external stress or stimuli may induce cell death and contribute toward the secretion of pro inflammatory molecules. The release of damage-associated molecular patterns (DAMPs) upon induction of therapy or cell death has been shown to induce an inflammatory response. Nevertheless, the mechanism as to how the DAMPs are released and engage in such activity needs further in-depth investigation. Interestingly, some studies have shown that DAMPs can be released through extracellular vesicles (EVs) and can bind to receptors such as toll-like receptors (TCRs). Ample pre-clinical studies have shown that cancer-derived EVs are able to modulate immune responses within the tumor microenvironment. However, the information on the presence of such DAMPs within EVs is still elusive. Therefore, this mini-review attempts to summarize and appraise studies that have shown the presence of DAMPs within cancer-EVs and how it affects the downstream cellular process.

## Introduction

Cancer has emerged as a significant issue globally, and it is now one of the main causes of mortality (1). The tumor microenvironment is heterogeneous consisting of cancer cells, stromal tissue, and the extracellular matrix (2, 3). Over the last few decades, the complex interaction between cancer cells and the host immune response has been extensively studied. The immune system plays a critical role in the tumor microenvironment, such as affecting cancer development and progression. One of the ways of eliminating cancer cells is by undergoing therapy such as chemotherapy, radiotherapy, or targeted therapy. Although some of these modalities have been proven effective, the after-effects of therapy may cause immunogenic cell death and eventually inflammation (4–7).

Cells undergoing cell death will secrete certain molecules into the environment that are immune-stimulating and may induce further inflammation (8–11). To survive, cells have a detection system that can sense possible danger and threats in their environment. In 1994, Matzinger (12) proposed the “danger” theory that cells can recognize and destruct danger when it is presented upon them without the need to distinguish self and non-self-threats (12). During an insult or intrusion, cells will release these endogenous molecules from within their compartment that is called damage-associated molecular patterns or DAMPs to alert the immune system (10, 13, 14). To note, PAMPs or known as the microbial pathogen-associated molecular patterns, such as formyl peptides or bacterial DNA, that are expressed by pathogenic microbes will also alert and activate the immune system (14). Likewise, dying cells also possess these “patterns” that act in a similar manner (10, 14, 15). These patterns coined, DAMPs can have different forms and be derived from various sources (14, 16). They can be expressed on the plasma membrane, be excreted extracellularly, or even be the breakdown products of certain pathways, and more recently, it can be found in extracellular vesicles (14, 17, 18). As such, this mini-review attempts to uncover some of the reported DAMPs derived from cancer-derived vesicles and how the downstream effects.

## DAMPs

DAMPs are molecules that are produced endogenously by cells in response to stress (19, 20). In cancers, high tumor apoptosis exerts stress and inflammatory signal that triggers the secretion of DAMPs leading to immunogenic cell death (ICD) of cancer cells (6). Unlike apoptosis, ICD is pro-inflammatory and requires the involvement of phagocytic immune cells such as dendritic cells (DC) and macrophages (4). It was found that the combination of apoptosis DAMPs secretion and ICD in response to specific anti-cancer agents such as chemo- and radiotherapy drugs could bring into play a potent and effective anti- tumor immunity, thus significantly becoming the target for cancer therapy (7, 21, 22). Krysko et al. defined DAMPs as molecules that perform non-inflammatory functions in living cells and acquire immune-modulatory properties when released on the cell surface (23). Work done by Apetoh et al. showed direct interaction of High molecular Group Box-1 (HMBG1), a well-known DAMP with Toll-like receptor-4 (TLR-4) on DCs that affect their antigenic presentation in breast cancer patients (24). On top of that, it was found that another type of DAMPs such as adenosine triphosphate (ATP) also plays a critical role in the degree of successful DC priming with cytotoxic T cells through NLRP3-dependent caspase-1 activation complex (25). As cancers are extremely adaptive, the failure of DAMPs to exert complete and effective anti-tumor response could also generate opposite mechanisms whereupon DAMPs are exploited by cancers to promote cancer growth and survival (26). As DAMPs could deliver effects on both anti-tumor and pro-tumor activities, the effort to decipher the molecular mechanism behind this is crucial.

## Extracellular Vesicles

Extracellular vesicles (EVs) are a class of membrane-encapsulated vesicles that are released by cells into the secretory system. Several types of EVs have been discovered depending on the biogenesis, function and size (27–29). According to the revised guidelines from International Society for Extracellular Vesicles (ISEV) committee, exosomes, or also known based on its size as small extracellular vesicles (sEVs), are a class of EVs derived from the formation of intraluminal vesicles that are usually sized between 30-100nm. Microvesicles, on the other hand, are a class of EVs that are released *via* the fusion of the cellular membrane and are usually larger than exosomes. Another class of EVs, called apoptotic bodies, are vesicles that are released by dying cells (27, 30). The heterogeneity of EVs has been one of the main limiting factors in understanding the role of EVs in cancer progression. Nevertheless, it has been shown by multiple studies that cancer-derived EVs can modulate the immune response through the regulation of immune cells such as CD8+ T cells, CD4+ T cells or natural killer cells (31). Upon response to therapy, cancer cells have shown to release a higher level of EVs (32). These EVs were shown to induce an immune response and may carry pro-tumorigenic cargo (32). Recently, DAMPs have been reported to be present within EVs and may affect the inflammatory balance within tumor sites. More importantly, some studies have shown that tumor-derived EVs are able to mediate toll-like receptor (TLR) signaling (33, 34). Since studies on DAMPs within EVs are limited, we will include all types of EVs including exosomes, small extracellular vesicles and microvesicles.

## DAMPs and EVs

### HMGB1 and EVs

Several immunostimulating molecules are discharged when cells die including HMGB1, uric acid, ATP and heat shock proteins (HSP) (14, 16, 35–37). HMGB1 is the most frequently encountered DAMP in cells undergoing stress (14). This protein, initially found in the 1970s, is a nuclear protein and binds to chromatin (5, 38). HMGB1 is highly conserved and is involved in various cellular processes such as DNA repair, gene expression and replication (39–42). Upon inflammation or cellular stress, HMGB1 binds to immune cell receptors such as Toll-like receptor 2 (TLR2), Toll-like receptor 4 (TLR4), and Receptor for advanced glycation end products (RAGE) (14, 43, 44). Apart from being released by cells infected with pathogens, dying cells or cells undergoing necrosis are also able to secrete HMGB1 (45). The release of HMGB1 may induce inflammation upon binding to different immune receptors through the release of cytokines such as tumor necrosis factor alpha (TNF-α) and interferon gamma (IFN-γ) (45). In cancer, the role of HMGB1 needs further understanding as dual roles of HMGB1 have been reported (42, 46). Pro-tumorigenic roles of HMGB1 include initiation of inflammation (47), enhance tumor cell proliferation (48), promotes tumor invasion and metastasis (49), enforces angiogenesis (50), involves in chemoresistance (51) and promotes antitumor immunity (52). Nevertheless, there have been reports that HMGB1 may also play a role as a tumor suppressor (53, 54). The paradoxical roles of HMGB1 in cancer have been of interest to many researchers over the years. What is more interesting, HMGB1 has been reported to be present in EVs as well. A study by Deng et al. showed that hepatocytes release HMGB1 *via* vesicles after being stimulated by lipopolysaccharide (LPS) (55). A follow-up study by the authors showed that HMGB1 was indeed packaged in exosomes and released extracellularly (56). The authors showed that HMGB1 was released in exosomes *via* the TLR4 pathway (56). In a different study, it was discovered that large burn injuries (LBI) were able to secrete plasma microvesicles enriched with HMGB1 (57). The study found that the released HMGB1 formed complexes with pro-interleukin-1-beta (pro-IL-1β) in both human and mouse plasma, and this heterocomplexes were able to induce immune dysfunction in LBI (57).

According to vesiclepedia (58), a database for protein/mRNA enriched in extracellular vesicles, HMGB1 is present in EVs such as exosomes and microvesicles from various sources. For instance, HMGB1 protein was reported present in EVs derived from breast cancer cells (59), bronchial epithelial cells (60), chronic lymphocytic leukemia cells (61), colorectal cancer cells (62, 63), glioblastoma cells (64), among others. These studies show that while HMGB1 is secreted as free HMGB1, this protein can also be secreted and packaged in vesicles as well. However, the type of EVs that contain HMGB1 has not been reported exclusively for one type of EV. The abovementioned studies show that HMGB1 can be present in exosomes, general extracellular vesicles as well as microvesicles. The exact mechanism as to how HMGB1 is sorted into these vesicles is still lacking information. The method of isolation of different types of EVs may also influence the presence of HMGB1 in these EVs. HMGB1 that is present within EVs has been shown to affect other surrounding cells as well. Functionally, several reports have also shown that EV-derived HMGB1 can participate in the carcinogenesis process. A study by Li et al. suggested that exosomal HMGB1 derived from esophageal squamous cell carcinoma managed to differentiate monocytes into the pro-tumorigenic Programmed cell death-bearing-tumor-associated macrophages (PD1+ TAMs) phenotype (65). A different study by Ye et al. showed that exosome-derived HMGB1 in hepatocellular carcinoma can activate B cells (66). This subsequently leads to the enhanced proliferation of TIM-1+ regulatory B cells by the TLR2/4 and Mitogen-Activated Protein Kinase (MAPK) pathways (66). Additionally, it was also shown that exosomal HMGB1 play a role in platelet-driven cancer malignancy. It was reported that treatment with anti-platelet drug, dipyridamole and aspirin inhibited tumor progression in Lewis lung carcinoma (LLC) cell lines and reduced the exosomal HMGB1 content. Similar finding was displayed in a tumor-bearing mouse model where combined treatment of dipyridamole and exosome-release inhibitor, GW4869 significantly mitigated tumor growth (67). Exosomal HMGB1 was also found to be involved in angiogenesis. A recent study by Gao et al. showed that hypoxic bone marrow mesenchymal cells were able to release exosomal HMGB1 that further enhanced angiogenesis *via* c-Jun N-terminal Kinase JNK/Hypoxia-inducible factor (JNK/HIF) pathway (68). It is interesting to note that the role of HMGB1 may differ depending on the form it is released. For instance, a study by Ma et al. showed that extracellular HMGB1 had opposing effects towards the expression of SAM and SH3 domain containing protein 1 (SASH1) as compared to exosomal HMGB1 (69). Although it is well known that extracellular HMGB1 is able to activate the inflammatory pathway *via* the TLR/RAGE receptors, information on EV-derived HMGB1 is still lacking and this calls for the need of further research. Since HMGB1 is a nuclear protein, it can be assumed that HMGB1 is packaged within EVs and may not be present on the surface, but further verification is needed. Therefore, the mechanism by which HMGB1 is able to stimulate immune response upon internalization of EVs still needs to be determined.

### HSP and EVs

Besides HMGB1, HSPs are commonly categorized as DAMPs as well (70). HSPs act as chaperones to ensure the proper folding of proteins (70, 71). These proteins are typically released when cells are under stress and are usually overexpressed in tumor cells due to the demand for cellular energy and the unstable environment (72). It was shown that certain HSPs trigger a pro-inflammatory response in mouse macrophage and human monocytes (73). Upon encountering HSPs, T regulatory cells (Tregs), T cytotoxic cells, natural killer (NK) cells, macrophages and DCs are activated (74). Nevertheless, the roles of HSP as DAMPs are still debatable. However, for the purpose of this review, we will consider HSPs as DAMPs and discuss the presence of HSPs in EVs. The presence of HSP-containing EVs released from cancer samples has been reported by several groups (75–80). For instance, a report by Gastpar et al. showed that HSP70 was present on the membrane of tumor-derived exosomes from pancreatic and colon cancer cell lines (81). The authors also showed that these exosomes were able to stimulate migration and HSP70 reactivity in NK cells (81). HSP70 has been reported to be released by tumor cells upon external stress such as radio or chemotherapy (77, 82). Therefore, it is presumed that under stressful conditions, the expression of HSP70 on exosomes is also increased. Lv et al. showed that there was indeed, a difference in the expression of HSP60, HSP70 and HSP90 in exosomes derived from HepG2 cells after treatment with chemotherapeutic drugs (83). Similar to the previous study, these HSP-containing exosomes were able to increase NK cell cytotoxic ability (83). A similar study by Elsner et al. suggested that HSP70-positive exosomes from melanoma cells were able to enhance NK cells cytolytic activity against YAC-1 cells (84). The increase of HSP70 in exosomes has also been shown upon induction by heat stress in murine models (85). Cho et al. showed that these heat-induced exosomes containing HSP70 elicit a stronger T helper type 1 (Th1) immune response (85). The presence of HSP70 in tumor-derived EVs has also been reported elsewhere. A study by Xie et al. showed that exosomes containing HSP70 stimulate anti-tumor immunity by enhancing the maturation of DCs and Th1 cells (86). A recent pilot study by Chanteloup et al. reported that exosomal HSP70 can be used to detect and monitor metastatic solid tumors such as breast and ovarian cancer (75, 76). HSP60 has also been shown to be released by tumor-derived exosomes (87, 88). A study by Wyciszkiewicz et al. showed that certain HSPs such as AlphaB-crystallin and HSP22 are present in exosomes from gynecological cancers (89). The authors showed that although these HSPs were present in both exosomes and serum, there is no correlation between the two sources (89). Similar to HMGB1, extracellular HSPs are not representative of exosomal/EV-derived HSPs in terms of abundance and function. According to vesiclepedia (58), HSPs, such as HSP90 were reported in cancer-derived EVs such as bladder cancer cells (90) and breast cancer (59). Almost all the reported studies show that HSPs are present within exosomes and not in other types of EVs. However, these studies report different techniques of isolation and characterization of exosomes and may not be conclusive enough to state that HSPs are exclusively found in exosomes. Nevertheless, though the presence of HSPs has been reported in EVs, the exact mechanism as to how these proteins induce an inflammatory/immune response is still elusive. The localization of HSPs as to whether it is present internally or on the surface of EVs warrants more studies (91). A study by Tang et al. showed that HSP90α is present on the surface of tumor-derived exosomes and is able to mediate communication with other cells (92). However, an earlier study by Clayton et al. showed that HSPs are also present in the lumen of exosomes and may not interact with target cells through cell surface receptors (93). Therefore, more in-depth studies are needed to determine whether HSPs are able to act as DAMPs and activate inflammation through certain receptors.

### S100 and EVs

S100 are a class of proteins known to bind to calcium and regulate intracellular and extracellular processes (94, 95). There are around 24 types of S100 protein members that can be divided into three main subclasses depending on their function (96). S100 proteins have long been recognized as DAMPs due to their ability to elicit an inflammatory response (97, 98). In cancer, the S100 proteins have been reported to be involved in carcinogenesis. In a study done by Hiratsuka et al., S100A8 and S100A9 proteins are found to be involved in lung cancer invasion and myeloid cell recruitment (99). S100 proteins have been reported to be present in EVs as well. A study by Prieto et al. showed that in chronic lymphocytic leukemia (CLL), S100A9 protein was present in the plasma exosomes (100). The authors showed that the exosomes containing S100A9 were able to activate the nuclear factor-kappa-light-chain-enhancer of activated B cells (NF-κB) pathway in leukemic cells (100). Not only that, in a different study by Li et al., the authors demonstrated that the S100A9 protein was also present in exosomes derived from follicular fluid of polycystic ovary syndrome patients (101). These exosomes were also able to promote inflammation *via* the NF-κB pathway (101). Although the molecular mechanism of the activation was still unmapped, this study, however, displayed an interesting finding in which the levels of NF-κB pro-inflammatory cytokines were increased upon incubation with S100A9-enriched exosomes (101). According to vesiclepedia (58), the presence of members from the S100 family was reported to be present in EVs from various sources. For instance, the S100A7A protein was found in EVs from colorectal cancer cell lines (62) and T cells (102). S100A5 protein was also found in colorectal cancer cell lines (103), and S100A12 protein was found in EVs from brain cancer cells, colorectal cancer cells, melanoma cells, kidney cancer cells and more (59). Similar to other DAMPs, the presence of S100 proteins is also not exclusive to one type of EV. Although the presence of S100 proteins has been reported in EVs, the actual function of S100 as DAMPs within EVs remains to be elucidated. Generally, free or extracellular S100 proteins are able to act as DAMPs by binding to receptors such as RAGE or TLR, but the mechanism of S100 within EVs still needs to be investigated. The presence of S100 proteins in EVs and how this affects the pathway leading to inflammation is still unknown.

### Micro RNA (miRNA)

**Figure 1:**
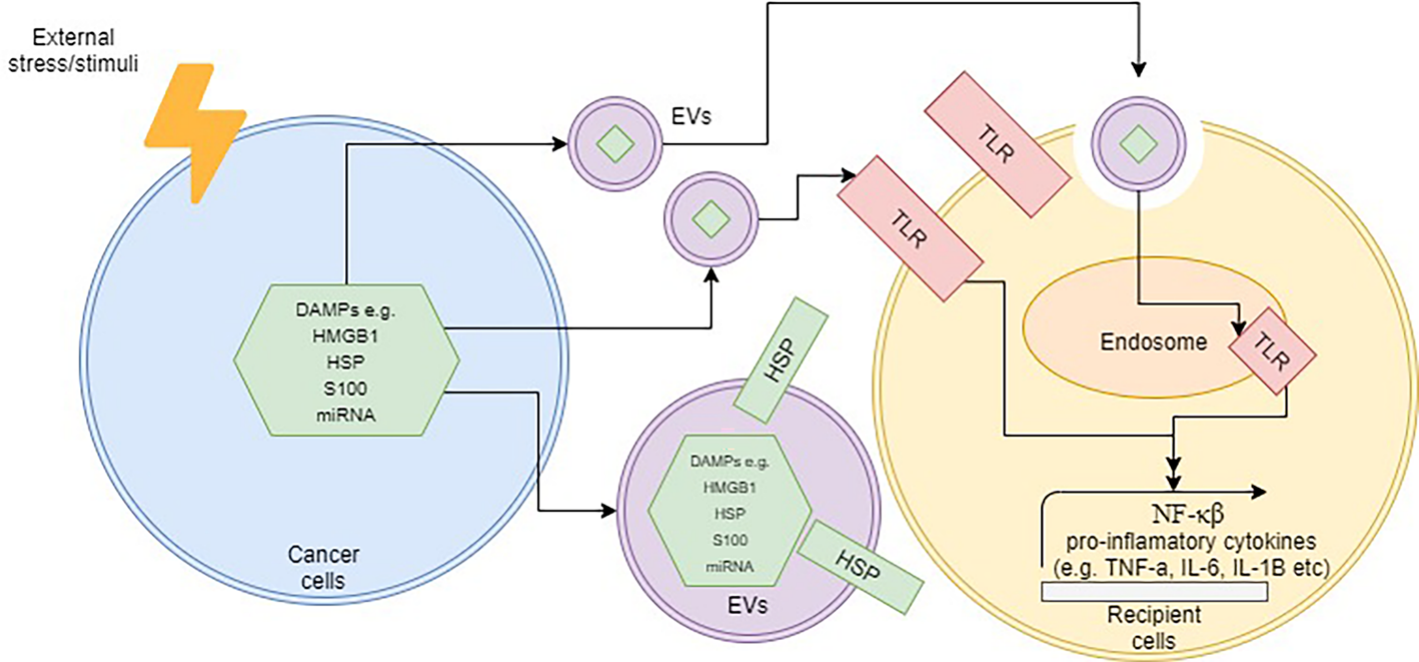
Schematic representation of how DAMP-containing EVs operate upon cellular stress. The localization of DAMPs can be internally or on the surface of EVs. Upon contact with target cells, the TLR pathway may be activated by DAMPs *via* the surface or endosomal route and subsequently trigger inflammation.

Besides the abovementioned molecules, other components within EVs that are also able to elicit an immune response is nucleic acid. It is well-established that microRNAs (miRNAs), short-lengthed nucleic aids, can be encapsulated within EVs. Some studies have shown that these EV-bound miRNAs were able to induce an immune response *via* the intracellular TLR pathway in several diseases (104–107). In rheumatoid arthritis, for instance, exosome-containing let-7b was able to differentiate macrophages into the M1 phenotype *via* TLR7 (108). A different study was able to show that miR-21 encapsulated in EVs was able to induce neurotoxicity through TLR7 signaling as well (109). In cancer, a study by Fabbri et al. demonstrated that exosome-derived miRNAs from lung cancer cells were able to bind to TLR8 on macrophages and activate the NF-κB pathway (110). This, in turn, led to the release of pro-inflammatory cytokines such as TNF-α and interleukin-6 (IL-6) (110). Although the presence of miRNA in EVs such as exosomes and microvesicles is well-established, there are still limited studies on whether these encapsulated miRNAs are able to stimulate TLR pathway, and subsequently activate inflammation. Additionally, most of the reported studies had purified EVs from sources that did not undergo any cellular stress such as chemotherapy or radiation, and thus the role of miRNA-EVs as DAMPs needs to be further determined. **Figure 1** demonstrates the overall schematic representation of how DAMPs are released within EVs and subsequently interact with target cells.

## Future Recommendations and Conclusion

Upon cellular stress or cell death, cancer cells will release a variety of molecules in response to the stimuli. Extracellular vesicles containing DAMPs have been hypothesized to induce an inflammatory response *via* the TLR/NF-κB pathway but are still in need of further verification. **Table 1** summarizes some of the reports that have shown the presence of DAMPs within EVs. However, most of these studies collect EV from samples that were not subjected to any treatment-induced stress. As such, we are not able to establish whether these DAMPs are significantly released upon stress or not. It has been well known that EVs released from cancer cells are able to modulate immune responses (31). Nevertheless, whether these modulations are induced through the regulation of DAMPs contained within the EVs remains to be elucidated. Additionally, little is known on whether DAMPs present in the EVs may induce the same response as free/extracellular DAMPs, and whether EVs provide more physiological benefits such as higher stability or longer half-lives. Additionally, the heterogeneity of EVs also plays a role in further understanding the role of EVs-DAMPs in inflammation. For instance, we are still unsure as to whether a certain subpopulation of EVs may carry certain DAMPs over other types of EVs. Most of the reported studies report either exosomes, extracellular vesicles and microvesicles as the source, which strengthens the fact that further studies are needed to determine whether DAMPs are secreted selectively. More importantly, the techniques used to isolate and characterize EVs such as exosomes vary from one study to another. It is imperative that studies pertaining to EVs adhere to the recommendation of the International Society of Extracellular Vesicles (ISEV) to ensure reproducible outcomes (111). Apart from that, the terminology used to describe EVs must follow the standards recommended by ISEV (111). Furthermore, the information on the localization of DAMPs within the EVs is also critical as this determines on which TLR or receptor is stimulated. Also, whether certain stimuli/therapy may induce the release of certain EV-derived DAMPs differently than the free DAMPs is still unknown. Most of the reported studies suggested that EV-derived DAMPs promote the pro-tumor environment. Nevertheless, the balance between pro- and anti- inflammatory and tumor responses regarding the release of DAMPs still needs further understanding. There are still some important questions that need to be answered in terms of the role of DAMPs within EVs, especially on how these molecules affect the tumor microenvironment and eventually cancer progression.

**Table 1:** A list of some of the reported studies that have shown the presence of DAMPs within EVs.

DAMPs	Type of EVs	Disease		Source		Reference
HMGB1	Exosome	Esophageal squamous cell carcinoma cell lines		Cell culture medium		(65)
	Exosome	Hepatocellular carcinoma cell lines		Cell culture medium		(66)
	Exosome	Lewis lung carcinoma (LLC) cell lines and mice model		Cell culture medium and blood		(67)
	Exosome	Glioma cells		Cell culture medium		(69)
HSP60, HSP70 and HSP90	Exosome	HepG2 hepatocellular carcinoma cells		Cell culture medium		(83)
HSP70	Exosome	Melanoma				(84)
HSP60 and HSP70	Exosome	NCI-H292 (human mucoepidermoid bronchial carcinoma), A549 (human lung adenocarcinoma) and K562 (human erythroleukemia) cell lines		Cell culture medium		(88)
HSP90	Extracellular vesicles	Bladder cancer cell lines		Cell culture medium		(90)
HSP90α	Exosome	Breast cancer cell lines		Cell culture medium		(92)
Alpha crystalline and HSP22	Exosome	Gynecological cancers		Serum		(89)
S100A9	Exosome	Chronic lymphocytic leukemia		Plasma		(100)
S100A9	Exosome	Polycystic ovary syndrome		Follicular fluid		(101)
S100A7A	Exosome	LIM1863 colon carcinoma cell-derived organoids		Cell culture medium		(62)
S100A5	Microvesicle	Colorectal cancer cells		Cell culture medium		(103)
S100A12	Extracellular Vesicles	Brain cancer cells, colorectal cancer cells, melanoma cells, kidney cancer cells		Cell culture medium		(59)
S100A4	Extracellular vesicles	Bladder cancer cell lines and bladder cancer patients		Cell culture medium and urine		(90)

## Author Contributions

NA conceived the idea. NA, NR, and SN contributed towards the writing of the manuscript. NA provided critical review and input. All authors contributed to the article and approved the submitted version.

## Funding

NAARB was funded by FRGS/1/2019/SKK08/UKM/01/2.

## Conflict of Interest

The authors declare that the research was conducted in the absence of any commercial or financial relationships that could be construed as a potential conflict of interest.

## Publisher’s Note

All claims expressed in this article are solely those of the authors and do not necessarily represent those of their affiliated organizations, or those of the publisher, the editors and the reviewers. Any product that may be evaluated in this article, or claim that may be made by its manufacturer, is not guaranteed or endorsed by the publisher.
